# Asynchronous Intermittent Regulation of Human Arm Movement with Markovian Jumping Parameters

**DOI:** 10.1155/2022/7848001

**Published:** 2022-11-15

**Authors:** Chao Ma, Wei Wu

**Affiliations:** ^1^School of Mechanical Engineering, University of Science and Technology Beijing, Beijing 100083, China; ^2^State Key Lab of Management and Control for Complex Systems, Institute of Automation, Chinese Academy of Sciences, Beijing 100190, China

## Abstract

In this paper, the regulation stability problem of the human arm continuous movement is investigated based on Markovian jumping parameters. In particular, the intermittent control mechanism is adopted in the arm movement regulation procedure to model the human intermittent motor control strategy. Furthermore, by taking into account the Markovian jumping parameters with different modes, the asynchronous regulation issue is proposed to model mode mismatch between the motor control and arm movement. On the basis of model transformation, sufficient stability conditions are established during the arm movements, and the desired regulation gain can be obtained by the convex optimization method. In the end, an illustrative example is presented to show the applicability and effectiveness of our developed model and optimized regulation approach.

## 1. Introduction

As a fundamental yet significant issue, the human arm movement regulation has been extensively studied over the last years with high interest. Especially, various computational theories with mathematical models have been developed to describe the human arm movements with corresponding motor control [1–3]. From the system point of view, the human motor system can be considered as a closed-loop control system that has complex dynamics and time-varying parameters. In fact, a simple arm movement is a complicated control procedure consisting of joint torques, external forces, and motor commands [4–6]. As a result, these dynamical models would lead to system analytical and synthetical complexities. Fortunately, different models of human arm movement control have been reported in the literature, where stability and trajectory tracking problems of movement regulation are effectively studied with theoretical results [7–9]. Furthermore, it is noted that most common computational methods of human arm movements are always embedded with constant parameters. As such, the optimal solution can be obtained by the computational method, which is dependent on precise model parameters. However, it should be pointed out that human arm control systems might have different control modes to achieve more adaptive abilities, and then, the activated muscles would change the shapes during the arm movements according to motion modes [10–12]. Hence, these jumping features can be further extended by the hybrid system models. In the meantime, Markovian jumping systems, as a special kind of hybrid system, have distinguishing modeling ability to depict the complex dynamic systems under structure or parameter changes [13–15]. The Markov chain has played a significant role in Markovian jumping systems to govern the mode jumpings. In fact, Markovian jumping systems have been widely studied to model robotic systems, neural network systems, and other control systems in numerous practical areas [16–20]. However, the control design difficulties would increase due to multiple subsystems under system mode information. More precisely, it is more applicable to the intrinsic variables within human arm movement control procedures. It is noticed that most simplified models have a common assumption on constant variable parameters, which have neglected the stochastic behaviors of intrinsic variables and then have certain model limitations. Therefore, employing many merits of theoretical analysis on Markovian jumping systems to the human arm dynamics can considerably help to improve the computational models and analysis methods. Moreover, it is worth mentioning that in practical applications, true system modes are always difficult to acquire, which gives rise to the so-called asynchronous control problems. Furthermore, the controller modes might be different from true system modes due to detection delays or external disturbances. Under this context, without considering the matching modes would lead to control performance degradation or even system unstable. As such, another interesting research topic lies in the fact of true mode information acquirement for synchronous mode-dependent control in practical implementations. By considering the observed modes and true modes, more robust mode-dependent controllers can be synthesized accordingly. As a result, it is natural and reasonable to further address the asynchronous regulation of human arm movements.

On the other hand, there are substantial evidences that have proven the intermittent strategies during human motor control [21–23]. More specifically, it can be found that human controls body movements in an intermittent way rather than continuous neural mechanisms [24, 25]. On the basis of this interesting feature, much effort has been devoted to the intermittent issues of human movements, and several mathematical models have been developed to exhibit the details. Generally speaking, by applying intermittent control, high efficiency and low cost can be achieved while the body operates movements [26, 27]. Under this context, intermittent control strategies can be employed for the human arm regulation problem from a system perspective. Moreover, the asynchronous intermittent control is more significant to deal with the multiple movement modes, which can further reveal the intrinsic dynamical characteristics. Very recently, intermittent control is also becoming a hot research topic in dynamical systems, and burgeoning investigations have been made towards the intermittent state estimation and stability control solutions, especially for the neural network systems [28–30]. From the control system perspective, minimal control cost is also an important issue for high-performance systems. As such, many in-continuous control strategies have been proposed, which include impulsive control, intermittent control, sampled-data control, event-triggered control [31–33]. Compared with continuous feedback control approaches, the intermittent control, as one of the notable discontinuous control strategies, can not only improve the control efficiency but also save the control cost. More precisely, by utilizing the nonzero control width, the intermittent control is more applicable for practical implementations. Furthermore, the intermittent control could be more conveniently formulated without event-triggering thresholds. However, to the best of the authors' knowledge, there are still few results on the asynchronous intermittent regulation of human arm movement with Markovian jumping parameters, which motivates us for this current study.

For the above discussions on the challenging and open areas, in this paper, the asynchronous regulation issue of human arm movement with Markovian jumping parameters is discussed, where the key idea is to mimic the motor intermittent control. Firstly, the mathematical model of human arm movement with Markovian jumping parameters is proposed. Secondly, the exponential regulation stability is analyzed with asynchronous intermittent control. Thirdly, sufficient stabilization criterion is derived, and the regulation synthesis is achieved via convex optimization. Finally, simulation results are provided to validate the usefulness and superiority of our theoretical results. This paper focuses on the theoretical investigations under the mean-square framework. Compared with major existing results, the main novelties of our paper can be summarized as follows:

1. A novel intermittent regulation model of human arm movement with Markovian jumping parameters is first proposed to mimic the dynamical human motor control mechanisms, which is presented in the state-space states with jumping modes for further regulation analysis and synthesis. The proposed hybrid model with jumping parameters is more applicable for practical implementations. It is noted that intermittent control for Markovian jumping control systems is also significant with exponential stability under the mean-square sense.
2. The asynchronous intermittent control scheme is presented to deal with the regulation stability under human motor and arm movement mode mismatch. In particular, the intermittent control strategy is proposed along with the practical asynchronous mode-dependent control scheme for the formulated Markovian jumping system. By utilizing the observed mode information instead of true modes, the mode-dependent regulation controllers can have more robustness for mode information acquirement. This can potentially improve the model's ability in describing the Markovian jumping regulation model.
3. A convex optimization approach is developed with sufficient linear matrix inequality conditions to ensure the mode-dependent regulation gain synthesis, and the corresponding illustrative simulation is performed to demonstrate the design adaptability. By solving the established conditions, the feasible solutions of desired mode-dependent regulation gains can be calculated accordingly.

Notations are as follows: *N* dimensional Euclidean space matrices are denoted by *ℝ*^*N*^. Matrix *ℙ* > 0 implies that *ℙ* is positive symmetric definite. (Ω, *𝔽*, *ℙ*) denotes a complete probability space. *𝔼*{·} stands for mathematics expectation. Finally, let *∗* denote the ellipsis terms in symmetry matrices and all matrices be algebraically compatible.

## 2. Problem Formulation and Preliminaries

### 2.1. Human Arm Movement Regulation Model

**Figure 1:**
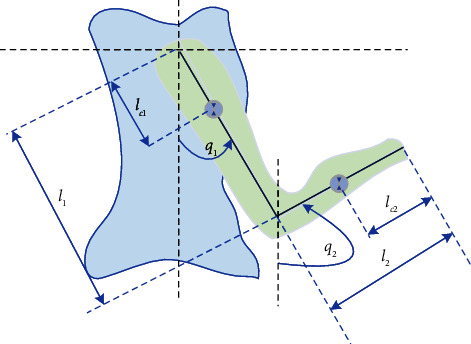
Illustration of the two-link human arm configuration.

As depicted in Figure 1, consider the following two-link human arm model with rigid motions:

(1) $$\begin{array}{c} M\left(q\right)\ddot{q}+C\left(q,\dot{q}\right)\dot{q}+G\left(q\right)=\tau \text{,} \end{array}$$

where

(2) $$\begin{array}{c} M\left(q\right)=\left[\begin{array}{cc} m_{1}l_{c1}^{2}+m_{2}l_{1}^{2}+I_{1} & m_{2}l_{1}l_{c2}\cos\;\left(q_{1}-q_{2}\right)\\ m_{2}l_{1}l_{c2}\cos\;\left(q_{1}-q_{2}\right) & m_{2}l_{c2}^{2}+I_{2} \end{array}\right]\text{,}\\ C\left(q,\dot{q}\right)=\left[\begin{array}{cc} 0 & m_{2}l_{1}l_{c2}\sin\;\left(q_{1}-q_{2}\right){\dot{q}}_{2}\\ -m_{2}l_{1}l_{c2}\sin\;\left(q_{1}-q_{2}\right){\dot{q}}_{1} & 0 \end{array}\right]\text{,}\\ G\left(q\right)=\left[\begin{array}{c} -\left(m_{1}l_{c1}+m_{2}l_{1}\right)g\;\sin\left(q_{1}\right)\\ -m_{2}l_{c2}g\;\sin\left(q_{2}\right) \end{array}\right]\text{.} \end{array}$$

More precisely, *q* denotes the joint displacement, *M*(*q*) denotes the inertia matrix, $C\left(q,\dot{q}\right)$ denotes the Coriolis and centripetal forces matrix, *G*(*q*) denotes the gravitational force vector, *τ* denotes the forces acting on the arm. Without loss of generality, it is assumed that the rotating cuboid feature is kept for the rotational links. Based on Lagrange's equation of motion in robotics, the two-link nonlinear dynamical model of the human arm is formulated to effectively describe the operating motions with multiple modes.

Consequently, in light of the fact that the muscles would change their shapes during arm motions, the Markovian jumping human arm model is then established to depict the jumping features of corresponding variations of the arm angular mass. Then, denote a continuous-time discrete-state Markov process *r*(*t*) in (Ω, *𝔽*, *ℙ*) to describe the jumping modes, where the transition probability matrix Π=(*π*_*ij*_)_*𝒩*×*𝒩*_, ∀*i*, *j* ∈ *𝒮*={1,…, *𝒩*} is defined by

(3) $$\begin{array}{c} \mathrm{Pr}\;\left(r\left(t+\Delta t\right)=j|r\left(t\right)=i\right)=\left\{\begin{array}{c} \pi_{ij}\Delta t+o\left(\Delta t\right),i\neq j\text{,}\\ 1+\pi_{ii}\Delta t+o\left(\Delta t\right),i=j\text{,} \end{array}\right. \end{array}$$

(4) $$\begin{array}{c} \pi_{ii}=-\underset{j=1,i\neq j}{\overset{N}{\sum }}\pi_{ij}\text{.} \end{array}$$

As a result, the linear Markovian jumping model can be obtained by the linearization method that

(5) $$\begin{array}{c} \dot{x}\left(t\right)=A_{r\left(t\right)}x\left(t\right)+B_{r\left(t\right)}u\left(t\right)\text{,} \end{array}$$

where $\left(t\right)={\left[{\dot{q}}_{1},{\ddot{q}}_{1},{\dot{q}}_{2},{\ddot{q}}_{2}\right]}^{T}\in R^{4}$ , *u*(*t*)=[*τ*_1_, *τ*_2_]^*T*^ ∈ *R*^2^ and *A*_*r*(*t*)_, *B*_*r*(*t*)_ are known weight matrices for certain system mode *r*(*t*).

**Remark 1 .**

The hybrid system models have been widely studied for human motion systems since the moment of inertia is influenced by human arm motions. In particular, as one kind of the important hybrid system, the Markov jump system can effectively model the complex dynamics under multiple operating modes. By introducing the Markov chain, different motion modes of the human arm can be depicted accordingly.

### 2.2. Asynchronous Intermittent Regulation Control

In order to mimic the concept of intermittent control strategy for human arm motions, the following mode-dependent intermittent regulation controller is synthesized with asynchronous features:

(6) $$\begin{array}{c} u\left(t\right)=\left\{\begin{array}{c} K_{\sigma \left(t\right)}x\left(t\right),t\in \left[k\right)T,kT+\left(\delta \right)\text{,}\\ 0,t\in \left[kT+\delta ,kT+T\right]\text{,} \end{array}\right. \end{array}$$

where *K*_*σ*(*t*)_ denotes the asynchronous feedback regulation gain matrix to be determined, *k* is the nonnegative integer, *T* represents the regulation control period, *δ* corresponds to the regulation control width with 0 < *δ* < *T*. Without loss of generality, *δ* and *T* are supposed to be prescribed setting parameters during the regulation design. Moreover, *σ*(*t*) ∈ Ϝ={1,…, *ℳ*} is the observable process of mode-dependent controllers, which has the same effect as *r*(*t*) and is related to *r*(*t*) with the following conditional probability representation Λ [34]:

(7) $$\begin{array}{c} \mathrm{Pr}\;\left\{\sigma \left(t\right)=\rho |r\left(t\right)=i\right\}=\lambda_{i\rho },\overset{M}{\underset{\rho =1}{\sum }}\lambda_{i\rho }=1\text{.} \end{array}$$

**Remark 2 .**

It is worth mentioning that the biological evidences have been reported to show the fact that the effective intermittent motor control is adopted by human muscle activations for energy-saving reasons. From the Markovian jumping system point of view, if the asynchronous mode mismatch is not fully taken into account, it would lead to certain control performance degradation or even system unstability. Furthermore, the asynchronous intermittent regulation control model is more applicable for Markovian jumping human arm systems by taking into account the possible neural-induced mode delays. This integrated scheme could improve the generality and applicability of the human arm control model to some extent.

Then, the resultant closed-loop human arm system can be obtained by

(8) $$\begin{array}{c} \left\{\begin{array}{c} \dot{x}\left(t\right)=A_{r\left(t\right)}x\left(t\right)+B_{r\left(t\right)}K_{\sigma \left(t\right)}x\left(t\right),t\in \left[k\right)T,kT+\left(\delta \right)\text{,}\\ \dot{x}\left(t\right)=A_{r\left(t\right)}x\left(t\right),t\in \left[kT+\delta ,kT+T\right]\text{.} \end{array}\right. \end{array}$$

Hence, the following mean-square exponential stability definition is presented to illustrate the equilibrium points:

**Definition 1 .**

[35, 36] The resultant closed-loop human arm system is said to be exponentially stable in the mean-square sense for any initial conditions *x*(0) if it holds that

(9) $$\begin{array}{c} E\left\{\left\|x\left(t\right)\right\|\right\}\leq ce^{-\lambda \left(t-t_{0}\right)}E\left\{\left\|x\left(0\right)\right\|\right\}\text{,} \end{array}$$

where *λ* > 0 and *c* > 0 are called the decay rate and decay coefficient, respectively.

Without loss of generality, it is denoted that *r*(*t*)=*i* and *σ*(*t*)=*ρ* for simplicity, respectively. To this end, the following matrix lemma is given for later use.

**Lemma 1 .**

[37] Given real matrices *𝒜*, *ℬ*, *𝒞*, *𝒳*, *𝒲*_1_, *𝒲*_2_ with appropriate dimensions, if there exists a positive symmetric *𝒫* satisfies that

(10) $$\begin{array}{c} \left[\begin{array}{ccc} PA^{T}+AP+X & B & PC^{T}\\ {}* & W_{1} & W_{2}\\ {}* & * & W_{3} \end{array}\right]<0\text{.} \end{array}$$

Then, there exist a positive symmetric matrix *𝒵* and a positive scalar *μ* > 0 such that

(11) $$\begin{array}{c} \left[\begin{array}{ccccc} -Z-Z^{T} & ZA^{T}+P & 0 & ZC^{T} & Z\\ {}* & -\mu^{-1}P+X & B & 0 & 0\\ {}* & * & W_{1} & W_{2} & 0\\ {}* & * & * & W_{3} & 0\\ {}* & * & * & * & -\mu P \end{array}\right]. \end{array}$$

## 3. Main Results

In this section, the theoretical, analytical, and synthetical results for closed-loop Markovian jumping human arm system regulation are derived with detailed mathematical proofs.

**Theorem 1 .**

Given constants *α* > 0, *β* > 0, *T* > 0, the closed-loop Markovian jumping human arm system can achieve the mean-square exponential stability with given mode-dependent regulation gains *K*_*ρ*_, *ρ* ∈ *ℱ*, if there exists a mode-dependent matrix *P*_*i*_ > 0, *i* ∈ *𝒮*, such that the following linear matrix inequality convex optimization conditions are feasible, where

(12) $$\begin{array}{c} \left\{\begin{array}{c} \alpha \delta -\beta \left(T-\delta \right)>0\text{,}\\ 2\alpha P_{i}+2P_{i}A_{i}+\overset{N}{\underset{j=1}{\sum }}\pi_{ij}P_{j}+2\underset{\rho =1}{\overset{M}{\sum }}\lambda_{i\rho }P_{i}B_{i}K_{\rho }<0\text{,}\\ -2\beta P_{i}+2P_{i}A_{i}+\overset{N}{\underset{j=1}{\sum }}\pi_{ij}P_{j}<0\text{.} \end{array}\right. \end{array}$$

**Proof**

Construct the following mode-dependent Lyapunov function as

(13) $$\begin{array}{c} V\left(x\left(t\right),i,t\right)=x^{T}\left(t\right)P_{i}x\left(t\right)\text{,} \end{array}$$

And define the infinitesimal operator *ℒ* for *V*(*x*(*t*), *i*, *t*) by

(14) $$\begin{array}{c} LV\left(x\left(t\right),i,t\right)=\lim_{\Delta ⟶0^{+}}\frac{1}{\Delta }\left\{E\left\{V\left(x\left(t+\Delta \right),j,t+\Delta \right)|\left(x\left(t\right),i,t\right)\right\}-V\left(x\left(t\right),i,t\right)\right\}\text{.} \end{array}$$

Consequently, for *t* ∈ [*kT*, *kT*+*δ*), it can be derived with respect to time that

(15) $$\begin{array}{c} LV\left(x\left(t\right),i,t\right)\\ =E\left\{\overset{N}{\underset{j=1}{\sum }}\pi_{ij}x^{T}\left(t\right)P_{j}x\left(t\right)+2x^{T}\left(t\right)P_{i}\dot{x}\left(t\right)\right\}\\ =\overset{N}{\underset{j=1}{\sum }}\pi_{ij}x^{T}\left(t\right)P_{j}x\left(t\right)+2x^{T}\left(t\right)P_{i}A_{i}x\left(t\right)+2\underset{\rho =1}{\overset{M}{\sum }}\lambda_{i\rho }x^{T}\left(t\right)P_{i}B_{i}K_{\rho }x\left(t\right)\text{.} \end{array}$$

Such that

(16) $$\begin{array}{c} LV\left(x\left(t\right),i,t\right)+2\alpha V\left(x\left(t\right),i,t\right)\\ \leq 2\alpha x^{T}\left(t\right)P_{i}x\left(t\right)+2x^{T}\left(t\right)P_{i}A_{i}x\left(t\right)\\ +\overset{N}{\underset{j=1}{\sum }}\pi_{ij}x^{T}\left(t\right)P_{j}x\left(t\right)+2\underset{\rho =1}{\overset{M}{\sum }}\lambda_{i\rho }x^{T}\left(t\right)P_{i}B_{i}K_{\rho }x\left(t\right)\text{,} \end{array}$$

where *α* > 0 is a constant scalar.

Then, it can be deduced by integrating the above inequality that

(17) $$\begin{array}{c} E\left\{V\left(x\left(kT+\delta \right),r\left(kT+\delta \right),kT+\delta \right)\right\}\leq E\left\{V\left(x\left(kT\right),r\left(kT\right),kT\right)\right\}e^{-2\alpha \delta }\text{.} \end{array}$$

If it holds that

(18) $$\begin{array}{c} 2\alpha x^{T}\left(t\right)P_{i}x\left(t\right)+2x^{T}\left(t\right)P_{i}A_{i}x\left(t\right)\\ +\overset{N}{\underset{j=1}{\sum }}\pi_{ij}x^{T}\left(t\right)P_{j}x\left(t\right)+2\overset{M}{\underset{\rho =1}{\sum }}\lambda_{i\rho }x^{T}\left(t\right)P_{i}B_{i}K_{\rho }x\left(t\right)<0\text{.} \end{array}$$

Similarly, for *t* ∈ [*kT*+*δ*, *kT*+*T*], one has that

(19) $$\begin{array}{c} E\left\{V\left(x\left(kT+T\right),r\left(kT+T\right),kT+T\right)\right\}\\ \leq E\left\{V\left(x\left(kT+\delta \right),r\left(kT+\delta \right),kT+\delta \right)\right\}e^{2\beta \left(T-\delta \right)}\text{.} \end{array}$$

If it holds that

(20) $$\begin{array}{c} -2\beta x^{T}\left(t\right)P_{i}x\left(t\right)+2x^{T}\left(t\right)P_{i}A_{i}x\left(t\right)+\overset{N}{\underset{j=1}{\sum }}\pi_{ij}x^{T}\left(t\right)P_{j}x\left(t\right)<0\text{,} \end{array}$$

where *β* > 0 is another constant scalar.

Hence, it follows that

(21) $$\begin{array}{c} E\left\{V\left(x\left(kT+T\right),r\left(kT+T\right),kT+T\right)\right\}\\ \leq E\left\{V\left(x\left(kT+\delta \right),r\left(kT+\delta \right),kT+\delta \right)\right\}e^{2\beta \left(T-\delta \right)}\\ \leq E\left\{V\left(x\left(kT\right),r\left(kT\right),kT\right)\right\}e^{-2\alpha \delta }e^{2\beta \left(T-\delta \right)}\\ \leq E\left\{V\left(x\left(kT-T+\delta \right),r\left(kT-T+\delta \right),kT-T+\delta \right)\right\}e^{2\beta \left(T-\delta \right)}e^{-\left(2\alpha \delta -2\beta \left(T-\delta \right)\right)}\\ \vdots \\ \leq E\left\{V\left(x\left(0\right),r\left(0\right),0\right)\right\}e^{-\left(2\alpha \delta -2\beta \left(T-\delta \right)\right)\left(k+1\right)}\text{.} \end{array}$$

Consequently, it can be verified that

(22) $$\begin{array}{c} E\left\{V\left(x\left(t\right),i,t\right)\right\}\\ \left.\leq e^{\frac{2\alpha \delta -2\beta \left(T-\delta \right)}{T}\delta }E\left\{V\left(x\left(0\right),r\left(0\right),0\right)\right\}e^{\frac{-\left(2\alpha --2\beta \left(T-\delta \right)\right)}{T}t},t\in \left[k\right)T,kT+\delta \right) \end{array}$$

and

(23) $$\begin{array}{c} E\left\{V\left(x\left(t\right),i,t\right)\right\}\\ \leq E\left\{V\left(x\left(0\right),r\left(0\right),0\right)\right\}e^{\frac{-\left(2\alpha \delta -2\beta \left(T-\delta \right)\right)}{T}t},t\in \left[k\right)T+\delta ,kT+\left(T\right)\text{,} \end{array}$$

which implies that

(24) $$\begin{array}{c} E\left\{V\left(x\left(t\right),i,t\right)\right\}\leq c_{0}E\left\{V\left(x\left(0\right),r\left(0\right),0\right)\right\}e^{\frac{-\left(2\alpha \delta -2\beta \left(T-\delta \right)\right)}{T}t}\text{,} \end{array}$$

with *c*_0_=*e*^2*αδ* − 2*β*(*T* − *δ*)/*Tδ*^.

Furthermore, by Lyapunov function *V*(*x*(*t*), *i*, *t*), it yields that

(25) $$\begin{array}{c} E\left\{V\left(x\left(t\right),i,t\right)\right\}\geq \lambda_{\min}\left(P_{i}\right){\left\|x\left(t\right)\right\|}^{2}, \end{array}$$

and

(26) $$\begin{array}{c} E\left\{V\left(x\left(0\right),r\left(0\right),0\right)\right\}\leq \kappa {\left\|x\left(0\right)\right\|}^{2},\kappa >0\text{.} \end{array}$$

Such that

(27) $$\begin{array}{c} E\left\{{\left\|x\left(t\right)\right\|}^{2}\right\}\leq \frac{\kappa c_{0}}{\lambda_{\min}\left(P_{i}\right)}e^{-\frac{\left(2\alpha \delta -2\beta \left(T-\delta \right)\right)}{T}t}{\left\|x\left(0\right)\right\|}^{2}\text{.} \end{array}$$

This means that the mean-square exponential stability can be satisfied according to Definition 1 and therefore completes the proof.

**Remark 3 .**

It should be pointed out that although multiple operation modes would affect the human arm motions, the mean-square exponential stability could ensure the fast convergence to the equilibrium points under the mode-dependent regulation control inputs. Especially, the conditional probability method for asynchronous feedback control is more applicable and efficient to deal with the parameter jumpings.

**Theorem 2 .**

Given constants *α* > 0, *β* > 0, *T* > 0, the closed-loop Markovian jumping human arm system can achieve the mean-square exponential stability if there exist mode-dependent matrices ${\tilde{P}}_{i}>0$, *i* ∈ *𝒮*, ${\tilde{K}}_{\rho }$, *ρ* ∈ *ℱ*, and matrix *Z*, such that the following linear matrix inequality convex optimization conditions are feasible, where

(28) $$\begin{array}{c} \left\{\begin{array}{c} \alpha \delta -\beta \left(T-\delta \right)>0\text{,}\\ \left[\begin{array}{cccc} -2\beta {\tilde{P}}_{i}+2A_{i}{\tilde{P}}_{i}+\pi_{ii}{\tilde{P}}_{i} & \sqrt{\pi_{i1}}{\tilde{P}}_{i} & \cdots & \sqrt{\pi_{iN}}{\tilde{P}}_{i}\\ {}* & -{\tilde{P}}_{1} & \cdots & \vdots \\ {}* & * & \ddots & 0\\ {}* & * & * & -{\tilde{P}}_{N} \end{array}\right]<0\text{,}\\ \left[\begin{array}{cc} \Theta_{i1} & \Theta_{i2}\\ {}* & \Theta_{i3} \end{array}\right]<0\text{.} \end{array}\right. \end{array}$$

With

(29) $$\begin{array}{c} \Theta_{i1}=\left[\begin{array}{cc} -Z-Z^{T} & \alpha Z+ZA_{i}^{T}+\pi_{ii}{\tilde{P}}_{i}+\underset{j=1}{\overset{M}{\sum }}\lambda_{i\rho }{\tilde{K}}_{\rho }^{T}B_{i}^{T}+{\tilde{P}}_{i}\\ {}* & -\mu^{-1}{\tilde{P}}_{i} \end{array}\right]\text{,}\\ \Theta_{i2}=\left[\begin{array}{cccc} Z & 0 & \cdots & 0\\ 0 & \sqrt{\pi_{i1}}{\tilde{P}}_{i} & \cdots & \sqrt{\pi_{iN}}{\tilde{P}}_{i} \end{array}\right],\\ \Theta_{i3}=\left[\begin{array}{cccc} -\mu {\tilde{P}}_{i} & 0 & \cdots & \vdots \\ {}* & -{\tilde{P}}_{1} & \cdots & 0\\ {}* & * & \ddots & 0\\ {}* & * & * & -{\tilde{P}}_{N} \end{array}\right]\text{.} \end{array}$$

Furthermore, when the above convex optimization conditions have feasible solutions, the desired asynchronous mode-dependent regulation gains can be determined by

(30) $$\begin{array}{c} K_{\rho }={\tilde{K}}_{\rho }Z^{-1}\text{.} \end{array}$$

**Proof**

Denote ${\tilde{P}}_{i}=P_{i}^{-1}$ and ${\tilde{K}}_{\rho }=K_{\rho }Z$ and perform matrix transformation manipulation to convex optimization conditions in Theorem 1. As a result, by applying Lemma 1, the rest of the proof can be straightforwardly obtained.

**Remark 4 .**

The established convex optimization conditions in Theorem 1 and Theorem 2 are in the form of strict linear matrix inequality, whose feasible solutions can be easily solved by Matlab or Yalmip software. Moreover, it should be addressed that the computational complexity is related to the matrix dimensions and mode information, which implies that the control design should effectively consider the model modes and model dynamical descriptions.

## 4. Illustrative Example

In this section, a simulation example is provided to validate the applicableness and effectiveness of the obtained results.

Consider the human arm model with two motion modes modification of operating equilibrium points as [*π*/2,0, *π*/2,0]^*T*^ and [0,0, *π*/2,0]^*T*^, respectively, and the following model parameters are chosen [38]:

(31) $$\begin{array}{c} m_{1}=1kg,m_{2}=2kg\text{,}\\ l_{1}=0.4m,l_{2}=0.4m\text{,}\\ l_{c1}=0.2m,l_{c2}=0.2m\text{,}\\ I_{1\left(r\left(t\right)=1\right)}=0.07kg/m^{2},I_{2\left(r\left(t\right)=1\right)}=0.017kg/m^{2}\text{,}\\ I_{1\left(r\left(t\right)=2\right)}=0.09kg/m^{2},I_{2\left(r\left(t\right)=2\right)}=0.018kg/m^{2}\text{,}\\ g=9.8\text{.} \end{array}$$

Then, the corresponding Markovian jumping model matrices can be obtained as follows:

(32) $$\begin{array}{c} A_{1}=\left[\begin{array}{cccc} 0 & 1 & 0 & 0\\ -0.0003 & 0 & 0.0003 & 0\\ 0 & 0 & 0 & 1\\ 0.0006 & 0 & -0.0006 & 0 \end{array}\right]\text{,}\\ A_{2}=\left[\begin{array}{cccc} 0 & 1 & 0 & 0\\ -0.0001 & 0.0326 & 0.0001 & 0\\ 0 & 0 & 0 & 1\\ 7.5990 & 0 & 14.1788 & 0.0071 \end{array}\right]\text{,} \end{array}$$

(33) $$\begin{array}{c} B_{1}=\left[\begin{array}{cc} 0 & 0\\ 6.0211 & -9.9317\\ 0 & 0\\ -9.9317 & 26.6915 \end{array}\right]\text{,}\\ B_{2}=\left[\begin{array}{cc} 0 & 0\\ 2.2222 & 0\\ 0 & 0\\ 0 & 10.1729 \end{array}\right]\text{,} \end{array}$$

where the transition rate for Markov chain of motion modes *r*(*t*) is assumed to be

(34) $$\begin{array}{c} \Pi =\left[\begin{array}{cc} -0.6 & 0.6\\ 0.4 & -0.4 \end{array}\right]\text{.} \end{array}$$

And *σ*(*t*) denoting asynchronous features is supposed to be with

(35) $$\begin{array}{c} \Lambda =\left[\begin{array}{cc} 0.7 & 0.3\\ 0.1 & 0.9 \end{array}\right]\text{.} \end{array}$$

By solving the convex optimization conditions in Theorem 2, the desired asynchronous mode-dependent control gains are obtained as follows:

(36) $$\begin{array}{c} K_{1}=\left[\begin{array}{cccc} -44.1239 & -117.7880 & 9.7824 & -75.8848\\ -18.5286 & -26.3105 & -25.4521 & -28.5790 \end{array}\right]\text{,}\\ K_{2}=\left[\begin{array}{cccc} -52.2699 & -132.9372 & -3.5499 & -68.7495\\ -20.8549 & -32.7957 & -29.7469 & -29.5057 \end{array}\right]\text{.} \end{array}$$

**Figure 2:**
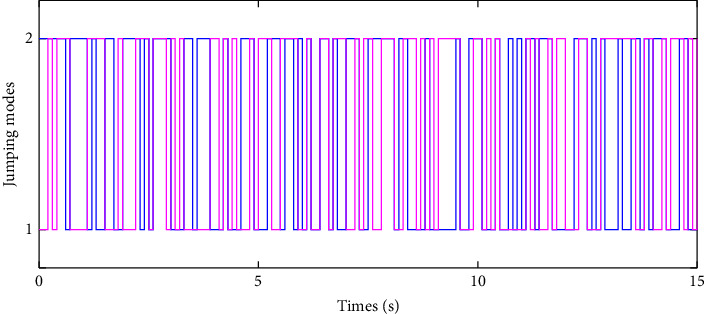
Illustration of the asynchronous jumping modes.

**Figure 3:**
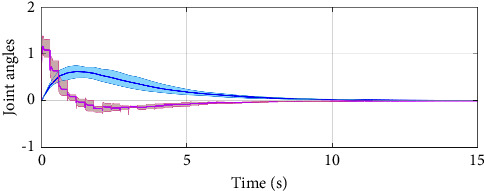
Illustration of the arm movement.

**Figure 4:**
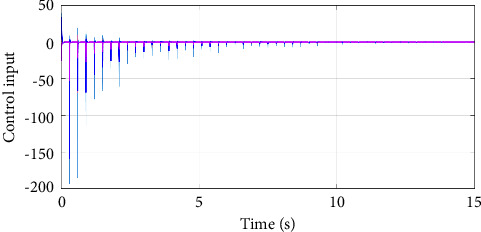
Illustration of the arm control input.

**Figure 5:**
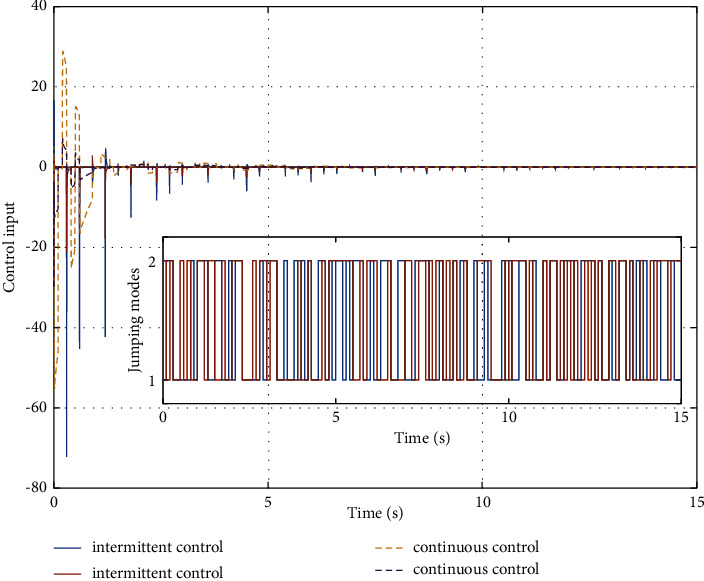
Comparison illustration of continuous control and intermittent control.

In the simulation, the simulation computer is with Intel Core i7 5 GHz processor, and the MATLAB software is utilized with variable-step configurations. The numerical parameters for the simulation model are based on the Monte Carlo method. For the intermittent control configuration, it is assumed that the regulation control period is *T*=0.3*s*, and the regulation control width is *δ*=0.1*s*. As a result, by setting zero initial conditions in the simulation, the jumping modes for the human arm model with asynchronous Markovian jumping mode features are depicted in Figure 2. With the prescribed simulation parameters, Figures 3 and 4 can show the closed-loop regulation arm motion trajectories and control inputs, respectively, where it can be found that our obtained asynchronous mode-dependent synthesized results can be well adapted to regulate the human arm movement under the intermittent stability control strategy.  Figure 5 shows the comparison results between asynchronous continuous control and our proposed asynchronous intermittent control strategies. It can be seen that the intermittent control can achieve the stable motion regulation with desired performance and less control cost, which also supports our established theoretical findings.

## 5. Conclusions

This paper is concerned with the human arm regulation control problem based on the established hybrid Markovian jumping system model. More specifically, the significant intermittent control strategy is explored by considering the fact of motor control mechanism. Furthermore, the asynchronous mode-dependent control features are employed to deal with the mode information mismatch by mode-induced delays. By means of convex optimization method, sufficient analysis and synthesis results are established. In the end, the applicability and effectiveness of our theoretical results are demonstrated via relevant simulation results. In future research, an important research is focusing on extending the current model to cases with more realistic motion constraints.

## Data Availability

All data supporting the findings of this study are available within the article.
